# Healthcare Cost Coverage and Hypertension and Diabetes Care Step Movement: A Five‐Year Follow‐Up Study in a Malaysian Semi‐Rural Community

**DOI:** 10.1002/hsr2.70740

**Published:** 2025-05-19

**Authors:** Adeola Folayan, Quek Kia Fatt, Mark Wing Loong Cheong, Tin Tin Su

**Affiliations:** ^1^ South East Asia Community Observatory (SEACO) and Global Public Health, Jeffrey Cheah School of Medicine and Health Sciences Monash University Malaysia Bandar Sunway Selangor Malaysia; ^2^ Global Public Health, Jeffrey Cheah School ofMedicine and Health Sciences Monash University Malaysia Bandar Sunway Selangor Malaysia; ^3^ Department of Pharmacy Practice, School of Pharmacy Monash University Malaysia Bandar Sunway Selangor Malaysia

**Keywords:** care step backward movement, care step progression, care steps, healthcare cost coverage, hypertension and diabetes

## Abstract

**Background and Aims:**

This study aims to understand how healthcare cost coverage (HCC) status affects hypertension and diabetes care across the three major care steps: awareness, treatment initiation and control.

**Methods:**

The probability of progressing the care steps was determined with logistic regression. The backward movements of two care steps (treatment and control) were investigated using McNemar's tests and presented with a Sankey diagram. All results were disintegrated by HCC status.

**Result:**

There was no evidence that having HCC contributed to any care step progression. However, there was no significant backward movement for diabetes treatment and blood pressure control for those with HCC, while those without HCC had a significant backward movement for diabetes treatment (54.3% [152/280], *p* < 0.001) and blood pressure control (31.6% [43/136], *p* = 0.04).

**Conclusion:**

Our results suggest that HCC supported a reduction in backward movement for some care steps but did not contribute to care step progression. HCC policies should aim to progress enrolees from awareness to initiating treatment and achieving control to attaining long‐term hypertension and diabetes control in low‐ and middle‐income countries.

## 1 Introduction

Non‐communicable diseases (NCDs) are known to be the leading cause of mortality globally [1]. They were previously viewed as a burden only in the high‐income countries but are now of concern in the low‐ and middle‐income countries (LMICs) [2]. Southeast Asia is said to have the highest increase in deaths due to NCDs [3]. NCDs account for 62% of all deaths in Southeast Asia, of which 50% of recorded deaths occur in people below the age of 70 [4]. NCDs are cardiovascular disease, diabetes, cancer and chronic obstructive pulmonary disorder [1, 5, 6, 7]. Diabetes is one of the leading causes of death in Malaysia [8]. About 15.6% and 29.2% of the Malaysian adult population live with diabetes and hypertension, respectively [8].

The management of hypertension and diabetes usually includes awareness (medically diagnosed), treatment initiation and control [9, 10, 11, 12]. The proper management of hypertension and diabetes can prevent or delay the onset of NCD‐related complications and mortality [13, 14]. Although, healthcare costs coupled with limited access to healthcare facilities pose substantial barriers to treatment‐seeking and management of NCDs for many families in the LMIC [2, 15]. However, healthcare cost coverage (HCC) is seen as a proxy for access to healthcare facilities, improving treatment seeking, healthcare utilization and health outcomes [16, 17, 18, 19, 20, 21]. Hence, HCC is expected to enhance the management of common NCDs such as hypertension and diabetes.

While HCC is an essential measure to improve the management of NCDs in LMICs, there remains a gap in the evidence of the role of HCC in each hypertension and diabetic care step. Many studies on NCD management are cross‐sectional and are unable to capture important elements in the care process, such as an individual's progression or backward movement on the care cascade [22]. For example, an individual might be diagnosed and aware of his status but fail to progress to the treatment care stage or discontinue treatment after treatment initiation. The treatment and control care stages are reversible, not permanent care steps [22]. An individual's circumstances might change due to some sociodemographic factors, which can influence their movement on the care step.

This study determined the distribution (in proportion) of adults with hypertension and diabetes who reported (i) having been previously diagnosed with hypertension and diabetes (aware), (ii) being treated for hypertension and diabetes, and (iii) having achieved blood pressure or blood glucose control. The association between HCC and each care step at baseline was determined. Furthermore, the probability of progression from (i) being hypertensive or diabetic and not aware to being aware, (ii) being aware to being treated or (iii) being treated to achieving control was also determined based on sociodemographic characteristics. The study also tracked the backward movement for two care steps (treatment and control) over 5 years. The backward movements occur, for example, when an individual moves from being treated to not treated or from achieving blood pressure or blood glucose control to having uncontrolled blood pressure or blood glucose levels. This is to understand which individuals by HCC status are likely to be lost and at what stage they are lost in the hypertension and diabetes management process. The evidence from this study will inform which stage of the care process needs restructuring and intervention. It will also guide policymakers on the necessary guidelines to be incorporated into the various HCC schemes to aid the effective management of hypertension and diabetes.

## 2 Methods

### 2.1 Data Source

The data is part of community health surveys in 2013 and 2018 by the South‐East Asia Community Observatory (SEACO) in Segamat, Johor, Malaysia. SEACO is a certified health and demographic surveillance system. Details of SEACO have been published previously [23].

### 2.2 Data Used and Study Design

This is a 5‐year longitudinal study. The data used for this analysis includes demographic data, data on HCC, blood pressure and blood glucose measurement. The data also contain information on hypertension and diabetes awareness, treatment and control.

### 2.3 Ethical Consideration

Participants provided written informed consent, and the study was approved by the Human Research Ethics Committee of the authors’ institute: (3837) for the Health Survey 2013 and (13242) for the Health Survey 2018.

### 2.4 Inclusion and Exclusion Criteria

**Figure 1:**
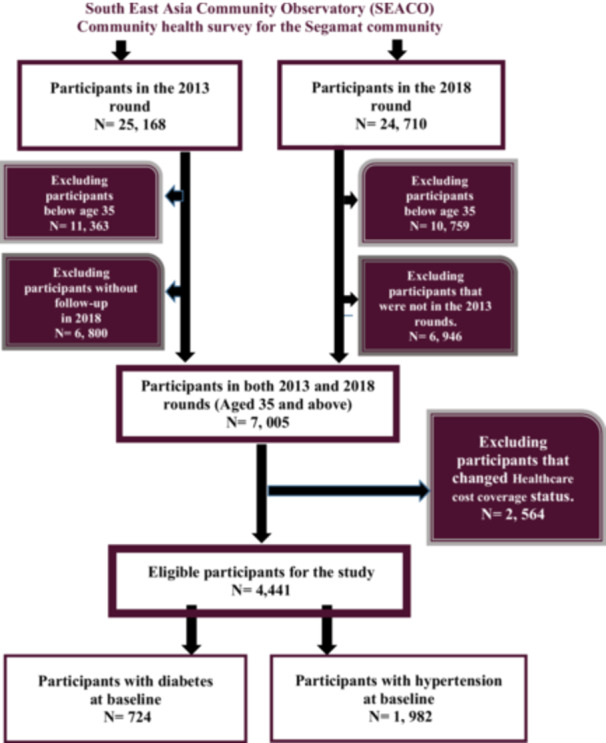
Flow chart of the study.

Participants were pulled from the 2013 and 2018 health rounds. Eligible participants were aged 35 and older, as SEACO only collects data on hypertension and diabetes for this age group. Those who were lost to follow‐up in 2018 were excluded from the study. Those who changed their HCC status from having HCC to not having HCC and vice versa were also excluded. Eligible participants met the criteria for being hypertensive or diabetic. The inclusion and exclusion flow chart is in Figure 1.

### 2.5 Demographic Variables

The demographic variables studied across the data set include age, gender, ethnicity, marital status, educational level, employment status, household income, and household size.

### 2.6 Independent Variable

For participants to be considered having HCC, they must have reported having any healthcare coverage plans such as government or pensioner insurance, employer‐provided insurance, private or personal health insurance, or employer or panel clinic coverage for healthcare costs. Those who make out‐of‐pocket payments by themselves, family or household members were considered not to have HCC.

### 2.7 Outcome Variables

The outcome variables are (i) being hypertensive or diabetic, (ii) hypertension and diabetes awareness, (iii) hypertension and diabetes treatment and (iii) hypertension and diabetes control. The cutoffs and definitions of the outcome variables are detailed in Appendix A.

### 2.8 Data Analysis

IBM SPSS Statistics, software version 27 and Microsoft Excel were used for data entry and analysis. Descriptive analyses were done to investigate the distribution (in proportion) of participants who achieved each care step. The difference in proportion was tested with a chi‐square test. The associations between HCC and each care step at baseline were determined using logistic regression. The probability of progressing each care step based on sociodemographic characteristics was also determined with a logistic regression. The regression models were adjusted for demographic variables. The group without HCC was the reference for coverage status. The backward movements of two care steps (treatment and control) were investigated with McNemar's tests and presented with a Sankey diagram created using SankeyMATIC. All results were disintegrated by HCC status. The adjusted odds Ratio (aOR) and 95% confidence interval (Cl) were used to report the size of the association. A *p*‐value < 0.05 was considered statistically significant. All statistical analyses were two‐sided.

## 3 Results

The baseline demographic characteristics of participants are shown in Tables 1 and 2.

**Table 1:** Baseline characteristics of participants with hypertension.

Variables		With healthcare cost coverage (*n* = 233) *n* (%)	Without healthcare cost coverage (*n* = 1749) *n* (%)	Total (*n* = 1982) *n* (%)
Age	35–44	12 (5.2)	186 (10.6)	198 (10.0)
	45–54	48 (20.6)	424 (24.2)	472 (23.8)
	55–64	78 (33.5)	693 (39.6)	771 (38.9)
	≥ 65	95 (40.8)	446 (25.5)	541 (27.3)
Gender	Female	116 (49.8)	1099 (62.8)	1215 (61.3)
	Male	117 (50.2)	650 (37.2)	767 (38.7)
Ethnicity	Malay	144 (68.0)	1131 (66.3)	1275 (64.3)
	Chinese	54 (18.8)	388 (20.2)	442 (22.3)
	Indian	27 (9.6)	207 (11.8)	234 (11.8)
	Others	8 (3.3)	23 (1.6)	31 (1.6)
Marital Status	Married	196 (84.1)	1424 (81.4)	1620 (81.7)
	Never married	3 (1.3)	46 (2.6)	49 (2.5)
	Divorced	3 (1.3)	18 (1.0)	21 (1.1)
	Widow(er)	31 (13.3)	245 (14.0)	276 (13.9)
	Others	0 (0.0)	12 (0.7)	12 (0.6)
	*Missing*		*4 (0.2)*	
Education	No formal education	21 (9.0)	53 (3.0)	74 (3.7)
	Primary	144 (61.8)	1283 (73.4)	1427 (72.0)
	Secondary	5 (2.1)	19 (1.1)	24 (1.2)
	Tertiary	9 (3.9)	13 (0.7)	22 (1.1)
	Others	54 (23.2)	349 (20.0)	403 (20.3)
	*Missing*		*32 (1.8)*	*32 (1.6)*
Employment status	Paid Employee	56 (24.0)	313 (17.9)	369 (18.6)
	Self‐employed	28 (12.0)	326 (18.6)	354 (17.9)
	House wife/husband	63 (27.0)	764 (43.7)	827 (41.7)
	Not working	44 (18.9)	231 (13.2)	275 (13.9)
	Pensioner	42 (18.0)	108 (6.2)	150 (7.6)
	*Missing*		*7 (0.4)*	*7 (0.4)*
Household income (RM)	B40 (< 3855)	227 (97.4)	1722 (98.5)	1949 (98.3)
	M40 (3856–8135)	5 (2.1)	25 (1.4)	30 (1.5)
	T20 (> 8135)	1 (0.4)	2 (0.1)	3 (0.2)
Household Size	< 4	130 (55.8)	878 (39.8)	1008 (50.9)
	= 4	27 (11.6)	259 (17.0)	286 (14.4)
	> 4	76 (32.6)	608 (42.7)	684 (34.5)
	*Missing*		*4 (0.2)*	

*Note:* USD 1 = RM 3.086 (01 June 2013).

Abbreviation: NCDs = Non‐communicable diseases.

**Table 2:** Baseline characteristics of participants with diabetes.

Variables		With healthcare cost coverage (*n* = 88) *n* (%)	Without healthcare cost coverage (*n* = 636) *n* (%)	Total (*n* = 724) *n* (%)
Age	35–44	7 (8.0)	59 (9.3)	66 (9.1)
	45–54	16 (18.2)	182 (28.6)	198 (27.3)
	55–64	33 (37.5)	260 (40.9)	293 (40.5)
	≥ 65	32 (36.4)	135 (21.2)	167 (23.1)
Gender	Female	44 (50.0)	404 (63.5)	448 (61.9)
	Male	44 (50.0)	232 (36.5)	276 (38.1)
Ethnicity	Malay	54 (61.4)	385 (60.5)	439 (60.6)
	Chinese	16 (18.2)	103 (16.2)	119 (16.4)
	Indian	16 (18.2)	143 (22.5)	159 (22.0)
	Others	2 (2.3)	5 (0.8)	7 (1.0)
Marital status	Married	70 (79.5)	520 (81.8)	590 (81.5)
	Never married	1 (1.1)	14 (2.2)	15 (2.1)
	Divorced	2 (2.3)	6 (0.9)	8 (1.1)
	Widow(er)	15 (17.0)	91 (14.3)	106 (14.6)
	Others	0 (0.0)	5 (0.8)	5 (0.7)
Education	No formal education	7 (8.0)	26 (4.1)	33 (4.6)
	Primary	57 (64.8)	457 (71.9)	514 (71.0)
	Secondary	3 (3.4)	10 (1.6)	13 (1.8)
	Tertiary	1 (1.1)	6 (0.9)	7 (1.0)
	Others	20 (22.7)	130 (20.4)	150 (20.7)
	*Missing*		*7 (1.1)*	*7 (1.0)*
Employment status	Paid Employee	23 (13.6)	118 (18.6)	141 (19.5)
	Self‐employed	6 (6.8)	100 (15.7)	106 (14.6)
	House wife/husband	27 (30.7)	279 (43.9)	306 (42.3)
	Not working	20 (22.7)	92 (14.5)	112 (15.5)
	Pensioner	12 (13.6)	44 (6.9)	56 (7.7)
	*Missing*		*3 (0.5)*	*3 (0.4)*
Household income (RM)	B40 (< 3855)	87 (98.9)	619 (97.3)	706 (97.5)
	M40 (3856–8135)	1 (1.1)	14 (2.2)	15 (2.1)
	T20 (> 8135)	0 (0.0)	3 (0.5)	3 (0.4)
Household size	< 4	47 (53.4)	307 (48.3)	354 (48.9)
	= 4	13 (14.8)	101 (15.9)	114 (15.7)
	> 4	28 (31.8)	226 (35.5)	254 (35.1)
	*Missing*		*2 (0.3)*	*2 (0.3)*

*Note:* USD 1 = RM 3.086 (01 June 2013).

Abbreviation: NCDs = Non‐communicable diseases.

### 3.1 Distribution of Individuals Across the Hypertension and Diabetes Care Steps in 2013 and 2018

**Figure 2:**
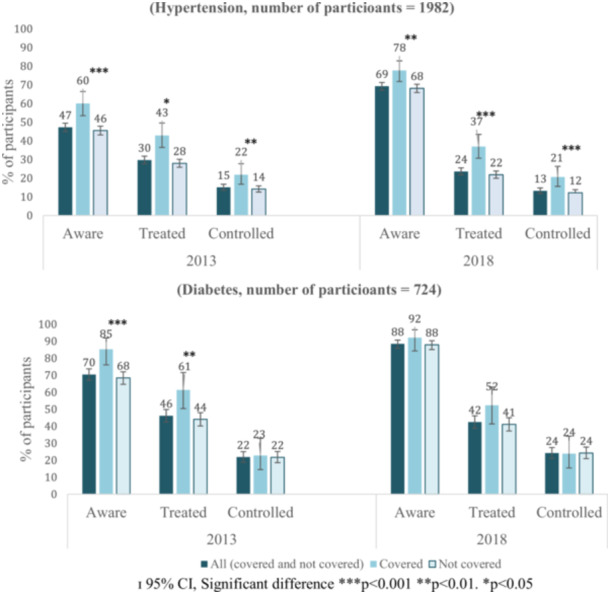
Distribution of hypertension and diabetes awareness, treatment and control among participants by healthcare cost coverage status in 2013 and 2018. ɪ 95% CI, Significant difference ***p < 0.001, **p < 0.01, *p < 0.05.

Compared with those without HCC, those with HCC had significantly higher proportions of participants who are aware of their hypertension status (*2013*: 60.1% (140/233) *vs.* 45.5%, (796/1749) *p* < 0.001 | *2018*: 77.7% (181/233) versus 68.2% (1192/1749), *p* = 0.003), being treated (*2013:* 42.9% (100/233) *vs.* 27.9% (489/1749), *p* = 0.03 | *2018:* 36.9% (86/233) versus 21.8% (382/1749), *p* < 0.001) and who had achieved blood pressure control (*2013*: 21.9% (51/233) *vs.* 14.2% (248/1749), *p* = 0.003 | *2018*: 20.6% (48/233) versus 12.2% (214/1749), *p* < 0.001) in both 2013 and 2018 (Figure 2). Similarly, those with HCC, compared with those without HCC, had significantly higher proportions of participants who were aware of their diabetes status (85.2% (75/88) *vs.* 68.4% (435/636), *p* < 0.001) and were being treated (61.4% (54/88) *vs.* 44.0% (280/636), *p* = 0.003) in *2013* (Figure 2). Participants with HCC had a higher proportion of participants who achieved all hypertension care steps but not all diabetic care steps.

### 3.2 The Association Between Healthcare Cost Coverage and Hypertension and Diabetic Care Steps at Baseline

Those with HCC had higher odds of achieving all care steps at baseline, except for blood glucose control (hypertension awareness: aOR = 1.666 (95% CI: 1.247–2.224) *p* < 0.001 | hypertension treatment: aOR = 1.795 (95% CI: 1.341–2.404) *p* < 0.001 | hypertension control: aOR = 1.443 (95% CI: 1.052–1.980) *p* = 0.02 | diabetes awareness: aOR = 2.595 (95% CI: 1.381–4.877) *p* = 0.003 | diabetes treatment: aOR = 1.970 (95% CI: 1.223–3.174) *p* = 0.005 | diabetes control: aOR = 0.550 (95% CI: 0.346–0.874) *p* = 0.01) (Table 3).

**Table 3:** Logistic regression of the association between healthcare cost coverage and hypertension or diabetes care steps in 2013.

	Hypertension		Diabetes	
	aOR (95%CI)	*p*‐value	aOR (95%CI)	*p*‐value
Awareness	1.666 (1.247–2.224)	< 0.001*	2.595 (1.381–4.877)	0.003*
Treatment	1.795 (1.341–2.404)	< 0.001*	1.970 (1.223–3.174)	0.005*
Control	1.443 (1.052–1.980)	0.02*	0.550 (0.346–0.874)	0.01*

*Note:* Outcome variables: Hypertension and diabetes awareness, treatment control.

Reference group: Without healthcare cost coverage.

Abbreviations: aOR, Adjusted odds ratio (sociodemographic data adjusted for includes age, gender, ethnicity, marital status, education and employment status); CI, Confidence interval.

* Statistically significant.

### 3.3 The Probability of Progression Based on Sociodemographic Characteristics

The proportion of participants that progressed the hypertension and diabetes care steps by healthcare cost coverage status are presented in the Supporting Information. HCC was not associated with all care step progressions except for the progression from being treated for diabetes without control in 2013 to having control in 2018 (aOR = 0.350 (95% CI: 0.124–0.987) *p* = 0.05) (Table 4). Surprisingly, this result shows that those with HCC are less likely to progress from being treated for diabetes without achieving blood glucose control to achieving blood glucose control. Hence, there was no evidence that HCC contributed positively to any care step progression.

**Table 4:** Logistic regression of the probability of progression based on sociodemographic characteristics.

Progression from being hypertensive or diabetic and not aware in 2013 to being aware in 2018				
	Hypertension awareness		Diabetes awareness	
	aOR (95%CI)	*p*‐value	aOR (95%CI)	*p*‐value
**Healthcare cost coverage**				
Without healthcare cost coverage (*Reference group*)				
With healthcare cost coverage	1.068 (0.690–1.652)	0.77	0.614 (0.187–2.018)	0.42
**Age**	1.017 (1.004–1.030)	0.01*	1.020 (0.987–1.054)	0.24
**Gender**				
Female (*Reference group*)				
Male	0.792 (0.573–1.097	0.16	0.863 (0.417–1.787)	0.69
**Ethnicity**				
Malay (*Reference group*)				
Chinese	0.863 (0.622–1.197)	0.38	1.642 (0.710–3.796)	0.25
Indian	0.819 (0.530–1.267)	0.37	2.554 (1.104–5.9.6)	0.03*
Others	1.363 (0.458–4.173)	0.57	0.752 (0.053–10.775)	0.83
**Marital status**				
Married (*Reference group*)				
Never married, divorced or widow(er)	0.904 (0.636–1.284)	0.57	1.143 (0.517–2.524)	0.74
**Education**				
Below Tertiary education (*Reference group*)				
Tertiary education	0.568 (0.312–1.034)	0.06	0.230 (0.056–0.949)	0.04*
**Employment status**				
Not employed (*Reference group*)				
Employed	0.847 (0.613–1.170)	0.31	0.614 (0.187–2.018)	0.42

Progression from being aware without treatment in 2013 to being on treatment in 2018.				
	Hypertension treatment		Diabetes treatment	
**Healthcare cost coverage**				
Without healthcare cost coverage (*Reference group*)				
With healthcare cost coverage	1.942 (0.919–4.100)	0.08	1.412 (0.550–3.626)	0.47
**Age**	0.989 (0.959–1.019)	0.46	1.024 (0.983–1.066)	0.06
**Gender**				
Female (*Reference group*)				
Male	1.645 (0.898–3.013)	0.11	0.679 (0.304–1.515)	0.34
**Ethnicity**				
Malay (*Reference group*)				
Chinese	1.986 (1.086–3.631)	0.03*	0.734 (0.274–1.963)	0.54
Indian	2.125 (1.072–4.215)	0.03*	2.237 (1.059–4.727)	0.04*
Others	0.000 (0.000–0.000)	> 0.99	1.872 (0.106–33.075)	0.67
**Marital status**				
Married (*Reference group*)				
Never married, divorced or widow(er)	0.927 (0.441–1.944)	0.84	0.433 (0.151–1.237)	0.12
**Education**				
Below Tertiary education (*Reference group*)				
Tertiary education	1.209 (0.362–4.038)	0.76	0.932 (0.199–4.369)	0.93
**Employment status**				
Not employed (*Reference group*)				
Employed	0.751 (0.401–1.408)	0.37	1.255 (0.560–2.813)	0.58

Progression from being treated without control in 2013 to having control in 2018				
	Hypertension control		Diabetes control	
**Healthcare cost coverage**				
Without healthcare cost coverage (*Reference group*)				
With healthcare cost coverage	1.082 (0.559–2.095)	0.82	0.350 (0.124–.987)	0.05*
**Age**	0.998 (0.969–1.029)	0.92	0.982 (0.941–1.023)	0.38
**Gender**				
Female (*Reference group*)				
Male	1.250 (0.699–2.237)	0.45	2.653 (1.122–6.270)	0.03
**Ethnicity**				
Malay (*Reference group*)				
Chinese	1.229 (0.703–2.147)	0.47	0.700 (0.263–1.863)	0.47
Indian	0.847 (0.367–1.956)	0.70	3.085 (1.252–7.601)	0.01*
Others	2.0136 (0.366–12.46)	0.40		
**Marital status**				
Married (*Reference group*)				
Never married, divorced or widow(er)	1.112 (0.562–2.199)	0.76	0.509 (0.179–1.443)	0.20
**Education**				
Below Tertiary education (*Reference group*)				
Tertiary education	2.107 (0.643–6.906)	0.22	2.015 (0.259–15.678)	0.50
**Employment status**				
Not employed (*Reference group*)				
Employed	1.339 (0.735–2.439)	0.34	0.557 (0.226–1.372)	0.20

*Note:* Outcome variables: Hypertension control and diabetes control.

Abbreviations: aOR, Adjusted odds; CI, Confidence interval.

* Statistically significant.

Although HCC did not affect most care step progression, sociodemographic variables that contributed to care step progression include age, ethnicity and education (Table 4). Being Indian ethnicity contributed to the three diabetes care step progression (from being diabetic and not aware to being aware (aOR = 2.554 (95% CI: 1.104–5.9.6) *p* = 0.03), from being aware and not treated to being treated (aOR = 2.237 (95% CI: 1.059–4.727) *p* = 0.04), from being treated without control to having blood glucose control (aOR = 3.085 (95% CI: 1.252–7.601) *p* = 0.01).

### 3.4 Hypertension and Diabetes Care Step Backward Movement

**Figure 3:**
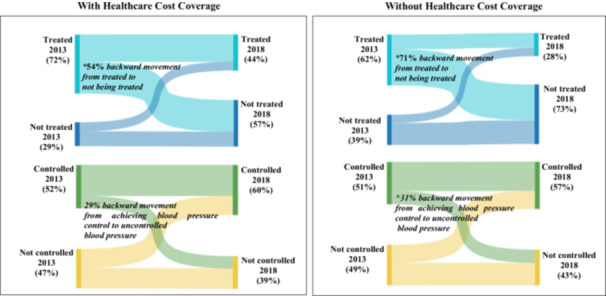
Care step backward movement in hypertension management by healthcare cost coverage status. *Significantly backward movement, p < 0.001. *^Significantly backward movement, p* = 0.04.

**Figure 4:**
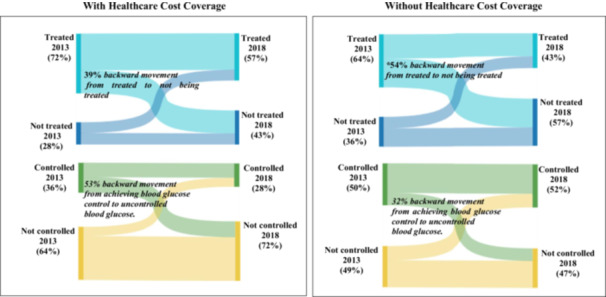
Care step backward movement in diabetes management by healthcare cost coverage status. *Significantly backward movement, *p* < 0.001.

Although, those with and without HCC had a significant backward movement from being treated for hypertension to not being treated. However, those with HCC had a lower proportion of backward movement compared with those without HCC (54.0% (54/100), *p* < 0.001 *vs.* 71% (346/487), *p* < 0.001). In addition, those without HCC had a significant backward movement for blood pressure control (30.6% (76/248), *p* = 0.04) and diabetes treatment (54.3% (152/280), *p* < 0.001), while those with HCC had no significant backward movement blood pressure control and diabetes treatment. HCC supports backwards movement reduction for most care steps (Figures 3 and 4).

## 4 Discussion

### 4.1 Main Finding of This Study

Participants with HCC had a higher proportion of participants who achieved all hypertension care steps but not all diabetic care steps at baseline. HCC was positively associated with all hypertension care steps at baseline but not all diabetic care steps. However, there was no evidence that having HCC contributed positively to any care step progression after 5 years. Ethnicity was associated with all diabetic care steps progressions. Those without HCC had a significant backward movement for diabetes treatment and blood pressure control, unlike those with HCC.

The proportion of participants with hypertension awareness was higher for those with HCC in 2013 and 2018 at 60.1% (140/233) and 77.7% (181/233), respectively, compared with those without HCC (45.5% (796/1749) and 68.2% (1192/1749) respectively). This agrees with a similar study among hypertensive participants in the United States; 82% of participants with private insurance had their blood pressure checked within 6 months, compared with 58% of the uninsured participants [24].

Likewise, more participants with HCC achieved hypertension control (2013: 21.9% (51/233) and 2018: 20.6% (48/233)) compared with those without HCC (2013: 14.2% [248/1749] and 2018: 12.2% [214/1749]). Although the proportion of participants with HCC that achieved hypertension control was only about 20% in this study, this proportion was nonetheless higher than the proportion reported in a study done in the United States, where only about 10% of the adult population with health insurance and usual health care source had blood pressure control (i.e., there were 90% with uncontrolled hypertension) [25].

Notably, the proportion of participants who achieved blood pressure and blood glucose control was generally low in this study, irrespective of the HCC status, ranging from 13% to 24%. This is not too far from the meta‐analysis of studies conducted in rural India, which stated hypertension control of 10.7% [26]. A similar study across Bangladesh, India, Indonesia, Malaysia, Viet Nam also reported that hypertension control was low across all study sites, ranging from 11.5% (189/1641; 95% CI: 10.0–13.0) to 20.4% (43/211; 95% CI: 14.9–25.8) [12]. However, this differs from an observation from a similar rural setting in South Africa, where nonphysician healthcare workers recorded 68% and 82% of blood pressure and blood glucose control, respectively [27].

Those with HCC had higher odds of being aware of their status at baseline (hypertension: aOR = 1.666 (95% CI: 1.247–2.224) *p* < 0.001, | diabetes: aOR = 2.595 (95% CI: 1.381–4.877) *p* = 0.003. This is related to a study among American adults, where PHI coverage was associated with decreased odds (0.82 (95% CI: 0.67–0.99) for unknown diabetes [28]. HCC is expected to improve hypertension and diabetes awareness. However, the effect of HCC on hypertension and diabetes awareness might be diverse. On the first note, the impact of HCC on hypertension and diabetes awareness largely depends on the HCC scheme offered to enrollees. e.g., PHI plans in Malaysia usually cover inpatient care but not outpatient care [29], similar to what is obtainable in India. This means that health insurance had a limited impact on hypertension screening [30]. Secondly, periodic interventions by government and nongovernment organisations might increase the awareness of NCD risk factors in general, like the implementation of the Komuniti Sihat Perkasa Negara (KOSPEN), the Healthy Communities, Building the Nation” program in Malaysia. Due to the high prevalence of hypertension and diabetes, the Ministry of Health implemented KOSPEN, a community‐based program, in 2013. This targeted NCD risk factors screening, patient‐centred care and referral, among others [4]. With the successful implementation of the programme, hypertension and diabetes awareness is expected to increase over the years, irrespective of HCC status.

HCC was positively associated with hypertension and diabetes treatment, similar to the reports in previous studies. Health insurance was reported to increase the likelihood of being on treatment for hypertension by 28.7% [31] and was positively associated with receiving diabetic treatment [19]. Uninsured and underinsured participants with hypertension were reported to be less likely to receive hypertension treatment compared to those with suitable health insurance [32]. This is expected as HCC is known to increase healthcare utilisation [16, 18].

Overall, this study showed that HCC is beneficial for hypertension control, similar to other studies [24, 33, 34, 35, 36, 37]. Duru et al. in their study, reported lower odds of blood pressure control among their study participants without health insurance (aOR: 0.63 (95% CI: 0.44–0.92) [24]. Another study by Oso et al. also reported a 2.2‐fold increased odds of achieving blood pressure control among patients with health insurance compared to those without health insurance [37].

HCC was negatively associated with the blood glucose control (aOR = 0.550 (95% CI: 0.346–0.874) *p* = 0.01). Although achieving blood glucose control has been positively related to having health insurance in previous studies [19, 38, 39, 40]. In Mexico, enrolment in Seguro Popular, a public health insurance plan known as People's Insurance, improved blood glucose control among diabetes patients, precisely poor adults [39]. However, one study reported that health insurance was not associated with blood glucose control among diabetic patients [41].

Current studies have shown that the rate of increase in hypertension and diabetic awareness, treatment and control in LMICs is generally lower compared with high‐income countries. Hypertension and diabetic diagnosis are convenient and should be prioritised in LMICs [10]. This could be achieved through different HCC schemes, as HCC was associated with better hypertension and diabetic care processes in most cases [42, 43, 44, 45, 46].

HCC was not related to the probability of progressing most care steps after 5 years. This suggests that HCC does not have efficient policies that facilitate hypertension and diabetic care step progression. However, ethnicity was associated with all diabetic care step progression. With regard to those of Malays ethnicity, those of Indian ethnicity are more likely to process the diabetes care steps. Policymakers should focus interventions on the ethnic groups most likely to be left out of diabetic care.

Those without HCC had more significant backward movement. Those without HCC might encounter financial barriers in initiating or continuing treatment to maintain blood pressure and glucose control. Unlike those without HCC, those with HCC might be propelled to continue treatment as long as the HCC plan that covers their treatment is still active. The health insurance industry should be explored as a means of retention in hypertension and diabetes care.

### 4.2 What Is Already Known on This Topic

No literature evidence has captured how HCC influence an individual's forward or backward movement in hypertension and diabetes care steps. Most studies have examined the association between HCC and hypertension and diabetes care steps. HCC was positively associated with better hypertension and diabetic care processes in most cases [42, 43, 44, 45, 46].

### 4.3 What This Study Adds

The evidence from this study suggested that HCC supported reductions in backward movement for some care steps, but it did not contribute to care step progression.

### 4.4 Limitation and Strength of This Study

This is the first study that assessed the effect of HCC on hypertension and diabetes care step backwards and forward movement. This study was done at a health and demographic surveillance system that only surveyed a geographically defined population. Hence, the result might not be a representation of the entire population.

## 5 Conclusion

HCC is beneficial for hypertension awareness, treatment and control. However, the effect of HCC on diabetic care is limited. HCC supports reduction in backward movement for most care steps, but it did not contribute to care step progression. HCC stakeholders and policymakers should ensure that HCC schemes have policies that facilitate care step progression. Hypertension and diabetes care step progressions are important to attaining long‐term hypertension and diabetes control in the LMIC.

## Author Contributions

**Adeola Folayan:** conceptualization, investigation, methodology, writing – original draft, validation, visualization, writing – review and editing, formal analysis. **Quek Kia Fatt:** methodology, validation, investigation, visualization, writing – review and editing, supervision. **Mark Cheong Wing Loong:** supervision, methodology, validation, visualization, writing – review and editing, investigation. **Tin Tin Su:** supervision, methodology, validation, visualization, writing – review and editing, conceptualization, investigation.

## Consent

The authors have nothing to report.

## Conflicts of Interest

The authors declare no conflicts of interest.

## Transparency Statement

The lead author Adeola Folayan affirms that this manuscript is an honest, accurate, and transparent account of the study being reported; that no important aspects of the study have been omitted; and that any discrepancies from the study as planned (and, if relevant, registered) have been explained.

## Supporting information

SuppMat.

## Data Availability

The data that support the findings of this study are available from the corresponding author upon reasonable request.

## Appendix A

Being hypertensive was defined as having a least 140 mmHg systolic and 90 mmHg diastolic blood pressure or being on blood pressure medication, based on the HEARTS guideline by the World Health Organization (WHO) [1] or being previously diagnosed with hypertension by a health worker. The first blood pressure measurement was not included in the analysis as recommended in the WHO HEARTS guideline [1]. Blood pressure level was determined by the mean of the second and third blood pressure measurements. Being diabetic was defined as having random blood glucose of 11.4 mmol or more [2], being treated by oral hypoglycemic agents or insulin, or being previously diagnosed with diabetes by a health worker.

Hypertension and diabetes awareness status was determined by the previous diagnosis. Participants were asked if a health practitioner had earlier told them they had hypertension or diabetes. Those who responded “yes” were considered aware of their status and are said to have known hypertension or diabetes. Hypertension and diabetes treatment were self‐reported as currently on blood pressure or blood glucose‐lowering medications. Hypertension control was defined as having less than 140 mmHg systolic and 90 mmHg diastolic blood pressure based on the mean of the second and third measurements, while diabetic control was described as having random blood glucose measurement of less than 11.4 mmol/L.

Participants must have achieved the previous care step to be eligible for inclusion in the next care step. That is, participants on hypertension or diabetes treatment must have been aware of their status. Similarly, participants who have achieved hypertension or diabetes control must have been aware of their status and being on treatment [1].

1. Geldsetzer P, Tan MM, Dewi FS, et al. Hypertension care in demographic surveillance sites: a cross‐sectional study in Bangladesh, India, Indonesia, Malaysia, Viet Nam. Bulletin of the World Health Organization. 2022;100(10):601.
2. Reidpath DD, Jahan NK, Mohan D, et al. Single, community‐based blood glucose readings may be a viable alternative for community surveillance of HbA1c and poor glycaemic control in people with known diabetes in resource‐poor settings. Global health action. 2016;9(1):31691.
