# Supplementary material for: Healthcare Cost Coverage and Hypertension and Diabetes Care Step Movement: A Five‐Year Follow‐Up Study in a Malaysian Semi‐Rural Community
Source: Health Sci Rep. 2025 May 19;8(5):e70740. doi: 10.1002/hsr2.70740 (PMC12086813; doi:10.1002/hsr2.70740)
Supplement: Supplementary file 1 — SuppMat. [file HSR2-8-e70740-s001.docx]

**Supplementary file**

Figure 1: The proportion of participants that progressed the hypertension care step by healthcare cost coverage status

Figure 2: The proportion of participants that progressed the diabetes care step by healthcare cost coverage status
